# Improving anticancer drug development begins with cell culture: misinformation perpetrated by the misuse of cytotoxicity assays

**DOI:** 10.18632/oncotarget.12673

**Published:** 2016-10-14

**Authors:** Alan Eastman

**Affiliations:** ^1^ Norris Cotton Cancer Center, Geisel School of Medicine at Dartmouth, Lebanon, NH, USA

**Keywords:** cytotoxicity assays, cell survival, apoptosis, target validation, synergy

## Abstract

The high failure rate of anticancer drug discovery and development has consumed billions of dollars annually. While many explanations have been provided, I believe that misinformation arising from inappropriate cell-based screens has been completely over-looked. Most cell culture experiments are irrelevant to how drugs are subsequently administered to patients. Usually, drug development focuses on growth inhibition rather than cell killing. Drugs are selected based on continuous incubation of cells, then frequently administered to the patient as a bolus. Target identification and validation is often performed by gene suppression that inevitably mimics continuous target inhibition. Drug concentrations *in vitro* frequently far exceed *in vivo* concentrations. Studies of drug synergy are performed at sub-optimal concentrations. And the focus on a limited number of cell lines can misrepresent the potential efficacy in a patient population. The intent of this review is to encourage more appropriate experimental design and data interpretation, and to improve drug development in the area of cell-based assays. Application of these principles should greatly enhance the successful translation of novel drugs to the patient.

## INTRODUCTION

The majority of anticancer drugs that enter clinical trials exhibit little or no therapeutic benefit and fail to obtain regulatory approval [1]. This high failure rate consumes billions of dollars annually, and contributes to the high cost of those few drugs that are eventually approved. There has been much discussion over the past few years about the rate of failure of novel anticancer drugs in clinical trials, and many reasons have been proposed including poor pharmacokinetics and drug bioavailability, unexpected toxicity, lack of efficacy, and regulatory hurdles [2, 3]. Others have placed the blame on the poor predictive value of preclinical models which do not effectively mimic human disease. Wilding and Bodmer [4] suggested “this has become such a widespread belief that it approaches dogma in the field of drug discovery and optimization, and has spurred a surge in studies devoted to the development of more sophisticated animal models.” As an example, it has been proposed that patient-derived xenografts (PDX) better predict clinical drug response, but the recently proposed use of 1000 PDX models for high-throughput drug screening is financially beyond the scope of almost every laboratory [5]. In fact, only 62 treatments were tested in these PDX models. Only a single dose/schedule was tested for each drug or combination, which may explain why the majority of treatments still failed to induce a significant response. Importantly, every drug tested had previously been developed in cell-based *in vitro* assays.

This review addresses a critical question: can we improve the preclinical development of drugs at an even earlier stage, before they reach animal and human testing? I believe that a major problem has arisen from inadequate and inappropriate preclinical evaluation of drugs, and a failure to place this development in the context of how the drugs will be administered to patients. This is an area that the pharmaceutical industry has under-funded; it has been estimated that only 7% of drug development costs are expended on preclinical research [2], yet an increased investment at this stage could reduce the exorbitant cost of failed clinical trials later. But the concerns apply equally to every academic laboratory involved in target identification and drug discovery. The rate of attrition of drugs in clinical trials could be reduced by better preclinical analysis, and only advancing truly promising drugs into clinical trials.

The ideal strategy to cure cancer is to kill all tumor cells while leaving enough normal cells alive that the patient survives. It is not surprising therefore that cytotoxicity assays have become a mainstay of cancer drug discovery. Unfortunately, there are many critical issues that are far too commonly ignored when using these assays. As a consequence, many inappropriate conclusions have been made that may have little if any relevance to the treatment of patients with cancer. Furthermore, this misleading information likely contributes to the failure of many drugs to effectively control cancer when tested in the clinical setting. The intent of this review is to encourage more appropriate experimental design and data interpretation. While the focus of the discussion, and the examples presented, is on traditional 2-dimensional cell culture, many of the concerns would equally apply to alternate cell culture models such as 3-dimensional spheroids that have been suggested as a better approximation of solid tumors.

## MOST “CELL VIABILITY” ASSAYS DO NOT MEASURE CELL VIABILITY

**Figure 1:**
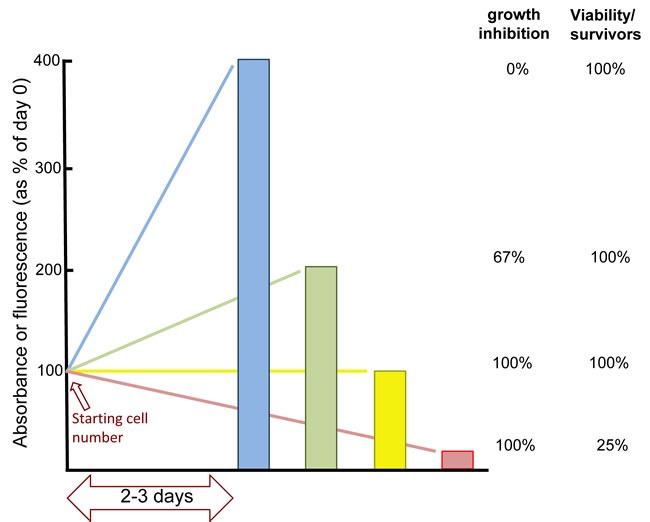
The misuse of cytotoxicity assays Because of its ease of application to multiple samples, and its low cost, tetrazolium dye reduction assays such as “MTT” or MTS” (available from many companies) are frequently used. This assay measures primarily mitochondrial dehydrogenase activity that is then extrapolated to reflect the number of cells in a culture dish. However, cells can rapidly change the activity of these enzymes such that it may not be an accurate reflection of the cell number. The CellTiter-Glo assay (Promega) relies on changes in ATP levels that can fluctuate rapidly with many environmental stresses so may not reflect the number of viable cells. Alternate assays measure total cell protein yet arrested cells can markedly increase their protein content without dividing, while dead cells still have protein. In our own experiments, we routinely quantify DNA content for high throughput assays as the possible variation per cell is generally limited to only 2 fold (i.e., whether the cells are in G1 or G2 phase of the cell cycle) [14, 21]. However, the major problem with all these assays it that they are almost ubiquitously referred to as viability assays when none of them measure cell viability. In an ideal situation where mitochondrial enzymes, ATP or DNA levels do not change per cell, these assays still only measure the number of viable cells. Consider a typical cytotoxicity experiment performed in a 96 well format. If you plate 1000 cells on day 1, the control may have 2000 cells on day 2 and 4000 cells on day 3 (blue line). If drug treatment results in 2000 cells on day 3 (green line), this is often reported as 50% viability even though it is an increase over the starting number of cells. To express the increase, it is necessary to subtract the starting cell number, so the increase from 1000 to 2000 actually reflects 67% growth inhibition (because the control increased by 3000). If the treated cells have completely arrested, there are still 1000 cells on day 3 (yellow line). In this case, the results will often be reported as “25% viability” even though there may be 100% growth inhibition and no loss of viability (albeit there is likely a mixture of dying and growing cells at this concentration). If the drug is not killing any cells, it will not cure the tumor, so a conclusion that only 25% of the cells are viable - implying 75% have died - would provide inappropriate optimism for a potential new therapeutic agent or strategy. If there were only 250 cells on day 3, this would indeed represent loss of viable cells (red line). However, this decrease in viable cell number can not be realized unless the starting number of cells is subtracted from the results. Unfortunately, few people measure the signal on day 1. Examples of curves from actual experiments of cell growth and death are presented in Figure 2.

Most cell-based drug screens use growth inhibition as an endpoint. The majority of studies use various commercial assays that are almost ubiquitously referred to as viability assays even though they do not measure cell viability (Figure 1). The term “viability” implies a measurement of both live and dead cells, and is an expression of the proportion that remain viable. Using these cytotoxicity assays, a reduction in signal by 50% compared to control usually means there are fewer cells; it does not mean that any cells have lost viability. Furthermore, if the rate of growth of a patient's tumor were decreased by 50%, it would still be called progressive disease. It is critical that preclinical development define concentrations and schedules that result in tumor cell killing if it is to translate to tumor shrinkage in the patient.

Tumor cell killing has alternately been measured by the ability of the cell to divide sufficiently to form a colony [6]. However, even colony formation assays have limitations as they reflect the number of cells that have proliferated to produce a colony of perhaps 50 cells within a given time frame (perhaps 14 days). But when counting the colonies, there are often smaller colonies and individual cells that may remain viable but whose proliferation was slowed or temporarily blocked by the drug. Consequently, these assays do not truly reflect only the surviving cells, but rather those that have been able to grow to a defined size within a defined time period. Cells that survive but do not form a colony within the time frame of the assay may mimic cells *in vivo* that could produce a relapse after initial response to therapy.

**Figure 2:**
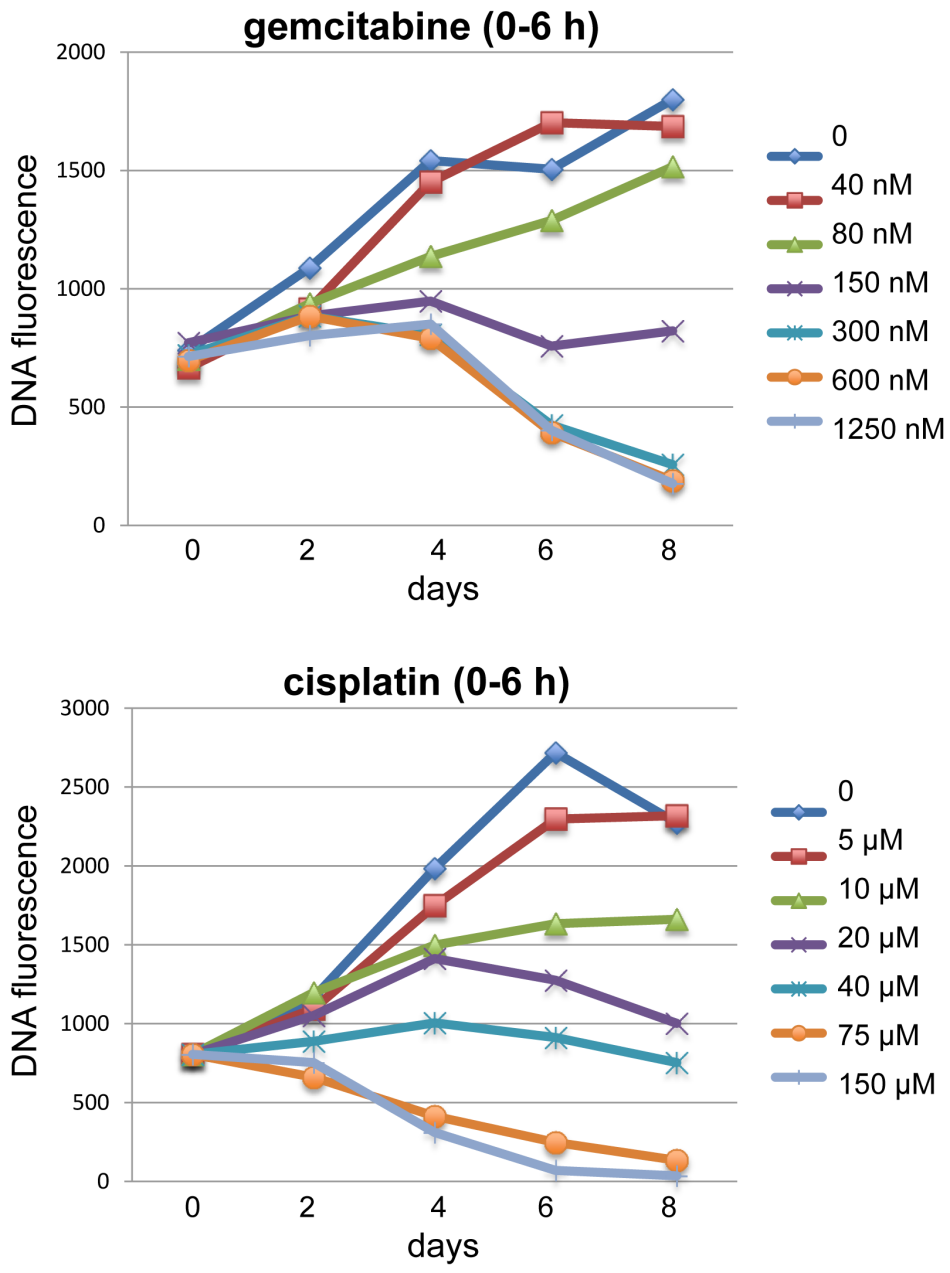
The impact of pulsed drug treatment on long-term cell growth and death Examples of long-term growth curves for cells incubated with either gemcitabine or cisplatin are shown. In both cases, asynchronously growing MDA-MB-231 breast cancer cells were incubated with drug at the indicated concentrations for 6 h, then the drug was removed, and the cells allowed to repair, grow, and/or die over the following 8 days. The experiment was performed in a 96-well format and DNA content was assessed at each time point [14]. By starting with sufficient cells/well, and harvesting a plate on day 0, the starting DNA content can be assessed. The growth rate of untreated cells is limited as the wells rapidly reach high cell density, and cells whose growth is partially inhibited will eventually attain the same cell number as controls. Cells incubated with either 150 nM gemcitabine or 20 - 40 µM cisplatin exhibit curves that would be considered “stable disease” in a patient. Higher concentrations of both drugs clearly caused a decrease in cell number, but this was not observed until 6 or 4 days following gemcitabine or cisplatin, respectively.

While colony formation assays may be closer to reporting cell killing, such assays are not generally as amenable to high-throughput drug screens as the modern commercial assays. Experimentally, it is possible to modify a growth inhibition assay so it can detect cell killing. This requires starting with more cells, and then assaying the decrease in cell number (or any surrogate marker of cell number) over time (Figure 2). Albeit, most treatments do not kill cells rapidly, so it is important to extend assays for a sufficient period of time. Using this approach, and plotting the results as percent survivors, the potential failure of many drugs would have been immediately evident.

There are alternate outcomes of drug treatment such as differentiation or senescence that can influence the apparent results of a cytotoxicity assay. In these cases, the results might be observed as 100% growth inhibition with no loss of cell viability. Appropriate assays exist to score these alternate endpoints if it is thought they might be occurring.

## INCUBATION TIMES IN VITRO OFTEN HAVE LITTLE RELEVANCE TO THE PATIENT

Most *in vitro* cytotoxicity assays use continuous incubation of cells with drug, yet many drugs are then administered to a patient as a bolus. *In vitro*, the continuous incubation limits recovery whereas in the patient, the tumor is only exposed to the drug for a short period followed by time for recovery, a scenario that is too frequently overlooked *in vitro*.

The first large drug screen was performed in the NCI60 panel of cell lines using a 2 day continuous incubation with each compound followed by a sulforhodamine B assessment of total protein [7]. By 1997, more than 60,000 compounds had been analyzed in this screen [8]. A more recent screen of 481 small molecules in 664 cell lines has been published, but a continuous incubation for 72 h was used, and the endpoint was total ATP level which was again misleadingly stated as being an assessment of viability [9]. A publically available database of the sensitivity of ~1000 cells lines to >100 drugs also exists (http://cancer.sanger.ac.uk/cosmic), but again relies on a 72 h continuous incubation with drug.

Most small molecules have a short half-life in patient plasma, but the consequence for target inhibition and cell killing varies with drug. Some drugs are rapidly inactivated or excreted while others may concentrate in the tissues. The binding affinity for the target also varies markedly. For example, some drugs irreversibly bind to their target (gemcitabine to ribonucleotide reductase; ibrutinib to Bruton's tyrosine kinase; afatinib to EGFR), some are pseudo-irreversible (5-fluorouracil to thymidine synthase; methotrexate to dihydrofolate reductase), while others have very high affinity for their target (e.g., vinca alkaloid binding to tubulin). These drugs can cause target inhibition long after any free drug has been cleared from the peripheral circulation. Most DNA damaging drugs covalently bind to DNA and require checkpoint activation, cell cycle arrest and DNA repair before recovery (or death). In contrast, many new targeted therapeutics are ATP mimetics that reversibly inhibit a protein kinase but rapidly dissociate from the target once the drug concentration decreases. In this latter case, it is usually critical to maintain chronic exposure to achieve continuous target inhibition.

Many new drugs may fail in the clinic because inadequate consideration has been given to how long a target needs to be inhibited. For readily reversible drugs, even daily administration to a patient may only inhibit its target transiently, permitting sufficient time for recovery each day. Hence, transient target inhibition may kill no tumor cells in the patient. Similarly, many drugs may only work at a particular phase of the cell cycle. Inhibitors targeting mitotic kinases are an excellent example [10]. The problem with targeting, for example, aurora kinase or PLK1 is that these kinases are only needed for a brief period of time during the cell cycle. Even if such a drug could quickly kill mitotic cells, the majority of cells in a tumor would still survive. Hence, such a drug would need to be present for an entire cell cycle if it is to elicit sufficient cell killing. In contrast, a drug that covalently modifies its target may persist until a critical phase of the cell cycle is reached.

Most of the current cytotoxicity screens camouflage these problems by continuous exposure of cells in culture, but as discussed, this is rarely how drugs are administered to patients. In preclinical development, cells need to be incubated with drug for various times (from a few hours to days), and then the ability to recover assessed. It should also be kept in mind that cells can arrest and survive for long periods of time, often for much longer than a typical cytotoxicity assay. Our experience suggests that recovery periods of at least 7 days should be investigated to determine whether cells die or recover (Figure 2). If continuous drug exposure is required for cell killing, then a continuous infusion or repetitive administration will be required. For example, “metronomic therapy” is based on the concept of giving low dose therapy on a repetitive, frequent basis. While this approach is often thought to target the tumor vasculature, it probably has significant impact directly on the tumor as well [11, 12]. For some drugs such as 5-fluorouracil or cytarabine, a daily or twice daily schedule is well established as the standard-of-care. Other approaches to provide extended drug exposure include liposomes that slowly release drugs over many days [13].

We recently published on the sensitivity of 65 cell lines to the Chk1 inhibitor MK-8776 [14]. The novelty of our screen was in using three different incubation times (24 h, 48 h or 7 day). Following the shorter incubation, drug was removed and cells allowed to grow until 7 days. Hence, the results after the short drug exposure reflect the ability of cells to recover. The three different incubation times provided very different results: about 15% of the cell lines were very sensitive to MK-8776 after only a 24 h incubation, 30% were sensitive after 48 h, while ~30% were completely resistant to continuous 7-day incubation with drug. These differences would have been completely missed if only a single incubation time had been used. These results suggest that a few tumors may be sensitive to a Chk1 inhibitor when administered as a bolus or short infusion.

A second example is the proteasome inhibitor bortezomib which has commonly been studied by continuous incubation in cell culture, yet a recent study has shown that a more clinically relevant 1-h incubation with bortezomib elicits much greater differential in sensitivity across myeloma cell lines [15]. A short treatment may also provide a much greater therapeutic index if it takes longer to kill normal cells. Hence, the time of exposure may provide an additional parameter that can enhance therapeutic index, and could be just as important as the administered dose. These examples highlight the need to perform extensive preclinical investigations to develop rationale schedules for effective drug administration to the patient.

## INCUBATION CONCENTRATIONS IN VITRO OFTEN HAVE LITTLE RELEVANCE TO THE PATIENT

Many publications justify their *in vitro* experimental protocol by stating that a drug is being used at a clinically relevant concentration, but in many cases this means that the experimental concentration approximates the peak concentration achieved in patient plasma. Unfortunately, this gives no consideration for the half-life of the drug *in vivo*, nor the amount distributed to the tumor. “Clinically relevant” has to mean much more than administration of a drug at a concentration that is only achieved briefly in patient plasma.

Consider the DNA damaging drug cisplatin as an example: the peak plasma concentration in patients is about 10 µM and its half-life is <1 hour [16]. A relevant design for an experiment with cisplatin would be to incubate cells with low µM concentrations for only a few hours, then remove the drug and follow the cells while they try, or fail, to recover. However, some *in vitro* experiments use concentrations as high as 100 µM and with incubations of 24-72 h [17]. At low drug concentrations, cisplatin works through cross-linking DNA. At these low concentrations, inhibition of transcription or protein function are not observed until cells are committed to die several days later [18]. These effects can be observed much earlier when higher concentrations are used, yet these concentrations and effects have no relevance to how the drug works in patients. Experiments at these high concentrations have simply created a clinically irrelevant science. Other examples of inappropriate drug concentrations *in vitro* have been addressed in a prior commentary [19].

There are other issues that make it difficult to extrapolate an *in vivo* concentration to an *in vitro* assay. For example, a drug may exhibit a long terminal half-life *in vivo* suggesting prolonged exposure of the tumor to drug. However, it is possible that the concentration during this terminal half-life is too low to effectively inhibit its target. In the case of the Chk1 inhibitor MK-8776, we note that the plasma concentration in patients drops below 1 µM by 6 h [20], and the lower concentrations thereafter are below those needed to inhibit Chk1 in cells [21].

Alternately, the concentration of drug in plasma may be high, and have a long half-life but this does not mean that the drug is bioavailable. Early studies with the non-selective Chk1 inhibitor UCN-01 established plasma concentrations that exceeded 20 µM (far higher than the 100 nM needed to inhibit Chk1) with a half-life of >200 h, but it was discovered that the drug was bound avidly to the plasma protein alpha-1-acid glycoprotein and hence was not bioavailable to the tumor [22, 23]. This problem was not predicted from studies with cell culture or animal experiments (it does not bind avidly to murine or bovine serum), but could have been discovered if human serum has been added to cell cultures [24].

Another issue that confounds extrapolation of *in vitro* concentrations to *in vivo* administration is the impact of drug metabolism that can result in drug inactivation. Even more important perhaps are the cases where metabolism is required to activate a pro-drug; examples include cyclophosphamide irinotecan, and nucleoside analogs (e.g., gemcitabine, cytarabine) that must be phosphorylated before they can impede DNA synthesis. Many of these metabolites only occur inside the tumor cells so their level cannot be assessed in blood. Consequently, pharmacodynamic analysis to assess the impact of target engagement in the tumor is required and this should always be included in the design of early phase clinical trials.

## CELLS CAN TAKE A LONG TIME TO DIE

Many years ago, we compared a variety of cytotoxicity assays following treatment of cells with cisplatin [25]. We noted a 10-fold range in GI50 values (50% growth inhibition) depending on the assay used, but critical to the current review is the fact that the cells took 4-6 days to die as assessed by trypan blue uptake. In a subsequent study, we observed that cells took 3-4 days to undergo apoptosis, consistent with the concentration and time for trypan blue uptake [26]. It is often feasible to kill cells much faster by using a higher concentration of drug, but this may involve a completely different mechanism that has no clinical relevance as discussed above.

In the case of DNA damaging agents, the delay in cell death (or apoptosis) is easy to reconcile. Cells initially perceive the damage, activate cell cycle checkpoints, arrest cell cycle progression, and attempt to repair the damage. At low levels of damage, cells may recover after a few days, which certainly contributes to the observed growth inhibition even though no cells may have died. When the level of damage is too high, cells may still progress slowly through the cell cycle and eventually reach a decision as to whether to undergo mitosis. The damaged cells appear to undergo a mitotic catastrophe and then apoptosis [27]. The mitotic catastrophe can occur prematurely in S phase-arrested cells, or after bypassing the G2 arrest [27–29]. We have performed cell cycle analysis to complement the growth inhibition curves shown in Figure 2. Concentrations of gemcitabine that killed cells were preceded by persistent arrest in S phase (data not shown), but whether they underwent mitotic events prior to dying remains to be determined.

The importance of this discussion is in highlighting that cells can arrest for a while before they die. The expectation that apoptosis, if it is going to occur, should be seen within 2 or 3 days has resulted in escalation of drug concentrations so that apoptosis occurs within a short time frame. I would recommend that one of the first assays to perform with any new agent is a long term analysis to assess the drug concentration and time over which cells die, perhaps using a simple assay such as trypan blue exclusion or measuring a decrease in the number of viable cells as in Figure 2. Only after it is established that cells die, and when, is it worth asking whether apoptosis was involved. There is certainly no rationale to study apoptosis if cells do not die.

Apoptosis provides many convenient assays, but they are often misused or the results misrepresented. Consider for example a caspase 3/7 enzyme assay (available from many commercial sources) where the results are obtained as optical density or fluorescence and then expressed as “fold increase in apoptosis.” The extent of apoptosis reported is primarily dependent on the base line value. If the base line apoptosis is only 1% of the cells, then a 5-fold increase as is typical for this assay might reflect only 5% of the cells undergoing apoptosis, leaving 95% of the cells alive [30–32]. Similarly, antibodies that selectively detect a caspase-cleaved substrate such as poly(ADP-ribose) polymerase (PARP) can be very sensitive, but provide no information whether the cleavage is occurring in a small percent of the cells or all of them [30, 33]. When analyzing PARP cleavage by western blotting, it is far more informative to use an antibody that detects both the parent and the cleaved form so as to be able to report the percent of cleavage [34, 35]. However, a further reservation is that when 50% of PARP is cleaved, it is still unknown whether this represents 100% cleavage in 50% of the cells, or 50% cleavage in 100% of the cells. The most informative assays for apoptosis record the percent of cells that are apoptotic. The most common assay uses Annexin V to measure the relocation of phosphatidylserine to the outer surface of the cell membrane. This assay is often called an early marker of apoptosis, yet it occurs fairly late in the apoptotic cascade as it is a consequence of caspase activity. We find that both PARP cleavage and chromatin condensation can occur many hours before the cells become positive for Annexin V [35, 36].

## TARGET IDENTIFICATION: GENE KNOCKOUTS MAY NOT PHENOCOPY DRUG INHIBITION

Genetic approaches are used frequently to identify and validate a target, and thereby provide the justification for a drug development program. Yet genetic knockdown or knockout can provide a very different result than transient inhibition of that protein. All proteins participate at some point in a protein:protein interaction, and many proteins function as a component of a multi-protein complex. If one protein is removed from this complex, the function of the other components can be unpredictable. One excellent example is the proteasome, wherein knockdown of one protein can cause failure of the other proteins to form a productive proteasome and consequently is lethal to the cells [37].

We have shown that suppression of the DNA exo/endonuclease Mre11 also reduces levels of Rad50 and Nbs1 because Mre11 stabilizes this protein complex [38]. In contrast, a small molecule inhibitor of Mre11 nuclease activity, mirin, does not change levels of these other proteins [39, 40]. Mre11 has several functions that are either dependent or independent of its nuclease activity [41] such that a small molecule inhibitor may have a very different outcome than genetic suppression.

A number of other examples have been reported where genetic analysis may mislead drug development. For example, it has been shown that an inhibitor of ATM does not phenocopy loss of the ATM protein [42]. Specifically, ATM inhibition prevented damage-induced sister chromatid exchange (SCE), whereas cells deleted of ATM exhibited normal SCE when damaged. Similarly, isoform selective inhibitors of the PI3K p110-beta can suppress tumor growth while deletion of the gene enhances tumorigenesis in mice [43]. This differential response is rationalized as a consequence of substitution by the p110-alpha isoform in the absence of p110-beta. It is unknown how many other examples may exist because such comparisons have been rarely made. It certainly warrants a cautionary note, particularly as more high throughput genetic approaches are being used as a starting point for drug development.

There is a further concern for all genetic suppression approaches used to identify novel targets as this strategy inevitably mimics continuous target inhibition and does not provide an opportunity for target recovery. As discussed above, this may have little relevance to how a drug is administered to a patient. As a consequence, a tremendous effort may be expended on what may eventually be deemed a poor drug target.

Finally, a limitation of knockout cells is the inability to generate any dose-response data, a property that is inherent with drugs. However, one valuable use of gene knockout systems is to assess the potential for off-target activities of a drug. If a cell lacking the target gene is still viable, then administration of a drug should have no impact on such a cell.

## CYTOTOXICITY IN CELLS MAY BE A CONSEQUENCE OF INHIBITION OF A DIFFERENT TARGET THAN EXPECTED FROM CELL-FREE ASSAYS

Most anticancer drugs are identified though a cell-free, *in vitro* assay; for example, kinase inhibition or disruption of protein:protein interactions. These drugs are then added to cells and, when inhibition of proliferation or cytotoxicity is observed, it is assumed the compound is working through the predicted target. However, this is a leap of faith that is too often not investigated further. In depth target validation experiments need to be performed in cell culture to ensure that the intended target is appropriately inhibited for the desired time. Target validation should also be confirmed in a patient once the drug enters clinical trials.

Our experience in this area relates to “BH3 mimetics” which target the BCL2 family of anti-apoptotic proteins. We have reported that the majority of putative BH3 mimetics do not inhibit BCL2 proteins directly in cells, but rather induce an integrated stress response that results in upregulation of the pro-apoptotic protein NOXA [36]. The consequence is selective inhibition of MCL1, another of the anti-apoptotic BCL2 proteins. These putative BH3 mimetics appear to function through a variety of different mechanisms, and often kill cells in a BAX/BAK-independent manner [36, 44–46]. Most recently we identified a novel pathway for the putative BH3 mimetic gossypol, demonstrating that it is an agonist of phospholipase A2, which as a consequence elevates intracellular calcium, endoplasmic reticulum stress and NOXA [47]. Several of these drugs are in clinical trials under the erroneous expectation that they act as direct inhibitors of BCL2 anti-apoptotic proteins (e.g., obatoclax, gossypol). While the induction of NOXA may provide a novel therapeutic approach, it is important to recognize the correct mechanism as it may inform novel therapeutic strategies and patient stratification. This problem of off-target effects of putative BH3-mimetics is addressed in more detail in a recent review [48].

The ability of a compound to inhibit its target in a cell-free system is often reported as IC50 (50% inhibition of activity) or Ki (affinity for target) and potency in the low nM range is commonly desired. An erroneous assumption is often made that this concentration of drug will also inhibit its target when added to cells. However, the intracellular concentration is usually much lower than the applied drug concentration because of extracellular protein binding, or because the cytoplasmic membrane can be an effective barrier between drug and target. The bioavailable concentration may also be limited by intracellular metabolism or protein binding. Generally a drug with nM inhibitory concentration *in vitro* will require µM concentrations to inhibit its target in cells. Hence, if a drug inhibits its target at µM concentrations in a cell-free system, as do many putative BH3 mimetics, and inhibits cell growth at the same concentration, then its proposed mechanism of action in cells should normally be considered suspect. Exceptions to this concern are drugs that are indeed concentrated in cells though usually into specific organelles such as weak acids in mitochondria or weak bases in lysosomes.

## OVER ENTHUSIASM FOR SYNERGY

If the combination of two drugs works better than either drug alone, we begin to get excited. If the combination is much greater than expected, we begin to think the interaction might be synergistic. We then apply a mathematical calculation to demonstrate that the drug combination is truly synergistic. But does demonstration of synergy in vitro really lead to clinically important advances? I believe the term synergy has been hijacked by mathematicians without understanding the critical biological questions. An extensive review of many mathematical approaches can be found in reference [49].

The most commonly used assessment of synergy is based on the median effect analysis whereby two drugs are tested at a constant ratio across a range of drug concentrations, starting with an equi-effective concentration; for example drugs are combined at a range of concentrations above and below the GI50. A combination index is then calculated and if it is <1, the drugs are deemed synergistic [50]. While this may be mathematically meaningful, it may have very little relevance to preclinical drug development. For example, a problem with medium effect analysis is that it supposes that drugs will be administered to patients at doses that will have a similar (median) effect.

As discussed above, most cytotoxicity assays only measure growth inhibition, yet as shown in Figure 2, higher drug concentrations may (hopefully) kill cells. An increase in cell death is the desired goal, but how does one express synergy when the endpoint changes? For example, if one combines two drugs that are equal in efficacy to 150 nM gemcitabine as shown in Figure 2 (i.e., 100% growth inhibition but no apparent cell death), the additive effect experimentally would be equivalent to that observed with 300 nM gemcitabine. In this case the number of viable cells decreased from 100% with 150 nM gemcitabine to about 20% with 300 nM gemcitabine after 8 days. If these were two different drugs, each eliciting 100% survival alone but with the combination resulting in 20% survival, it would be considered synergistic according to current mathematical models, yet the experimental design clearly shows only additivity. Perhaps a better definition of synergy might be when the effect of the drug combination exceeds that achieved simply by increasing the concentration of either drug alone by two fold. The magnitude of the effect also depends on the time point at which measurements are made as the cell killing is more evident at longer times.

As growth inhibition is the usual endpoint of most cytotoxicity assays, most synergy assessments are performed at lower, sub-optimal concentrations of each drug, so that a combination effect can be observed. If one uses two drugs that each inhibit cell growth by 95%, it would not be possible to calculate a combination index. In fact, synergy is most commonly observed at drug concentrations eliciting low efficacy as single agents. This is evident in a recent investigation that combined gemcitabine and a Chk1 inhibitor [51]. The authors compared three different calculations of synergy (Bliss, Lowe and Highest single agent) and reported that synergy was observed at sub-GI50 concentrations of each drug. Another recent example is the reported synergy between inhibitors of Her2 and CDK4/6; the problem with this study is that incubation with a CDK4/6 inhibitor should arrest the cells in G1, yet its failure to do so demonstrates that it was not (or minimally) inhibiting its target [52]. Hence, I question whether a combination of drugs at sub-optimal concentrations has any real relevance to treating the patient who will hopefully be administered optimal doses.

It has been stated that synergism is independent of mechanism of action because the mass-action law-based determination of synergism is mechanism independent [53]. Unfortunately, the mechanism of action of two drugs can have a critical impact on the experimental design and conclusions. This is particularly evident with drugs that perturb the cell cycle where one drug may arrest the cell cycle and thereby elicit resistance to the second drug; this would be deemed antagonism. There is a classic example of the cell cycle kinetic effects associated with the combination of vinblastine and cytarabine in a murine leukemia model [54]. When both drugs were given simultaneously, they were antagonistic. However, as the time between treatments was extended an additive or even synergistic effect was observed. These observations can be explained if vinblastine treatment transiently synchronized the cells in mitosis, so that 16 h later, there were many more cells progressing through S phase to be susceptible to cytarabine. A similar cell kinetic association was observed with the combination of paclitaxel and cisplatin wherein greater efficacy was observed when cisplatin was administered after paclitaxel [55, 56]. Hence, mechanistic understanding of each drug should lead to a rationale schedule of administration. It should also be emphasized that these experiments were performed *in vivo* in which the drugs were cleared by the mouse such that the tumor was only exposed to each drug for a short time period. Experiments using continuous treatment schedules *in vitro* would have been unable to observe this effect.

CPX-351, a mixture of cytarabine and daunorubicin in a slow-release liposomal formulation, is an interesting exception to the apparent need for appropriate drug scheduling. The molar ratio of the two drugs (5:1) was derived from *in vitro* synergy experiments using continuous drug treatments [57]. The slow release liposome circumvents the different clearance rates for the two drugs, and maintains this molar ratio *in vivo*. Improved therapeutic responses have been observed in patients with AML [13]. As these studies did not assess the potential role of cell cycle kinetics, it is possible that an even greater therapeutic response might be attainable with different drug schedules.

An understanding of the mechanism of action of each drug in a combination is critical for another reason. Consider a Chk1 inhibitor that inhibits its target but does not elicit any cytotoxicity as a single agent, as we have shown for many cell lines [14]. If the concentration of the drug is escalated to achieve a GI50 concentration, it is probable that the cytotoxicity is due to inhibition of some other target. While the drug might still be inhibiting (or killing) more cells, it is no longer relevant to the target and hypothesis being tested. If a drug inhibits its target, its concentration should not be increased further, and the outcome of the drug combination should perhaps be referred to as an enhancement ratio such as the fold decrease in GI50 when the second drug is added. These situations are perhaps much more exciting than situations in which both drugs are cytotoxic.

## THE FOCUS ON ONLY A FEW CELL LINES CAN MISREPRESENT THE EFFICACY OF A DRUG

Despite the recent analysis of large panels of cell lines in a few publications as discussed in Section 2, the majority of cancer biology still tends to focus in depth on one cell line, and then only occasionally compares outcomes in a few other lines. Such experiments miss the variability that exist across cell lines and that could lead to the identification of novel therapeutic strategies in hypersensitive subsets of patients. One relevant example has been the extensive use of the U2OS osteosarcoma cell line to study DNA damage-induced checkpoint regulation [58, 59], yet this cell line turns out to be one of the few that is hypersensitive to Chk1 inhibition [14, 21], and consequently, the different responses to Chk1 inhibitors has been missed. Put another way, the overt use of one sensitive cell line may lead to the expectation that a drug should have broad therapeutic activity in patients.

The potential variation in drug responses has been primarily driven by clinical observations. For example, early clinical trials with EGFR inhibitors did not recognize that tumors with a gain-of-function mutation in EGFR would be uniquely sensitive [60]. Additional preclinical analysis may have identified this sensitivity and saved millions of dollars in the cost of clinical trials by selecting only those patients with the greatest likelihood of responding. These days all patients are screened for mutations in EGFR prior to prescribing such therapy, and a similar approach will need to become the standard for all new targeted therapeutic strategies.

Another example from clinical observations is a patient with metastatic bladder cancer whose remarkable response to the mTOR inhibitor everolimus was attributed to a loss-of-function mutation in TSC1, a suppressor of the mTOR pathway [61]. It is suggested that mutated TSC1 may be present in 9% of bladder cancers and so may provide a responsive patient population. Positive response to everolimus has also been reported in patients with sub-ependymal giant cell astrocytomas, a disease associated with inactivation of TSC [62].

A more extreme case is the one patient who had a complete and durable response to the topoisomerase inhibitor irinotecan plus the Chk1/Chk2 inhibitor AZD7762. Genomic sequencing led to identification of a mutation in RAD50, a component of the ATM-dependent DNA damage response pathway, and subsequent experiments validated this mutation as being causative in the sensitivity of the tumor [63].

Clinical trials may identify these outlier responses, yet these trials would be far less expensive if potential responders were identified before the trial began. We discussed above our screen for cell lines sensitive to Chk1 inhibitors, and discussed the availability of other large databases that may predict sensitivity to drugs. But I reiterate my concern that most of those screens have used continuous drug treatments that may provide results that are overly optimistic compared to a patient receiving bolus drug treatment. The detection of potential responders may require appropriate assays across large panels of cell lines, but that will still be far cheaper than conducting a large clinical trial in which very few responders are seen.

## TUMOR HETEROGENEITY AND A SOLUTION FOR IT WAS RECOGNIZED 50 YEARS AGO

Tumor heterogeneity is synonymous with drug resistance. The realization that tumors contain multiple genetic variants, exhibit a heterogeneous phenotype and consequently contain clones with different responses to drug is not a new observation [64, 65]. However, even before this realization, drug treatments had been developed to circumvent the problem. The earliest administration of chemotherapeutic drugs involved single agents, but it rapidly became evident that drug combinations were much more effective. A series of guidelines were developed, the most important of which for the current discussion is that multiple drugs that do not have overlapping mechanisms of resistance should be combined (certainly they should avoid overlapping toxicities too) [66]. Many standard therapies involve 3-6 different drugs while in the case of childhood acute lymphocytic leukemia, curative therapy has involved administration of up to 11 different drugs through remission induction, consolidation and maintenance therapy [67]. The new era of targeted molecular therapeutics has unfortunately reverted primarily to administration of single agents. Resistance to single drugs is inevitable, and the overt optimism that one new molecular targeted drug will cure cancer needs to be discarded. Most of these new drugs provide only marginal increase in life span, and at an enormous expense [68] (imatinib and crizotinib are possible exceptions). Unfortunately, the goal of clinical trials has taken a backward step to accept an “increase in life-span, ” but the dream of a cure is still possible if we build effective multi-drug combinations.

A palpable tumor has >10^9^ tumor cells, though most advanced tumors present with far more cells. Given a spontaneous mutation frequency at any locus of 10^-6^, there will already be at least 1000 cells resistant to any drug at the time of presentation or relapse (usually many more as the mutation frequency in a tumor with genomic instability is thought to be much higher). Indeed, mutations eliciting resistance to targeted therapies have been identified in biopsies collected prior to therapy [69, 70]. One problem in clinical trials is that we wait until the tumor has reappeared before initiating a second line of therapy, yet resistance to that therapy will already have developed. Chemotherapy (which includes targeted therapies) would be much more effective if administered when there is minimal disease. Debulking (surgery) is one means to reduce tumor load, as is commonly used in breast cancer patients, and is then followed by adjuvant therapy even if there is no detectable tumor. Yet surgical options are often limited, detection of tumor margins difficult, and micrometastases missed. I believe we should not discard the well-established chemotherapy agents that have been the mainstay of therapy for many decades, and are still the most effective drugs in use. While these drugs have only cured some cancers, they are often effective initially at reducing the tumor burden. Consequently, we should be developing therapeutic strategies that combine surgery and established chemotherapy (or radiation), and novel targeted therapies should be administered when the tumor burden is minimal.

I discussed the issue of whether drug combinations should be considered synergistic. In designing drug combinations to circumvent resistance, it will be important to recognize that any drugs that are only effective in combination will increase the number of possible resistance mechanisms, and resistance to either drug would make the combination ineffective. Consequently, it will be far more beneficial to combine two drugs that have completely independent actions, and independent mechanisms of resistance. Any drugs that are only effective in combination should be considered as a single drug when adding up the total number of drugs in the combination.

There is one final issue in developing drug combinations that has rarely been addressed. Cell culture-based research generally operates on the concept that the two drugs work on a single cell. *In vivo*, this rationale does not have to hold, and drugs may target different cells, for example tumor cells at different phases of the cell cycle, or the microenvironment or vasculature. Furthermore, drugs do not have to be administered simultaneously but could be administered in alternating or sequential schedules. This might reduce the potential increase in toxicity which can result in having to decrease the dose with a concomitant decrease in efficacy.

Today, we can dream of true precision medicine where tumor DNA is sequenced to predict which drugs will be most effective against a particular tumor. Tumors have multiple mutations, and hopefully many Achilles heels, and hopefully many drugs can be identified that target these susceptibilities. If these drugs are selective for the tumor, then it should be possible to combine them without increased toxicity and thereby provide effective combination therapy.

## SUMMARY

The efficacy of a drug in a clinical trial is usually recorded as stable disease, partial or complete remission. These endpoints are far removed from most of the preclinical research in which growth inhibition has been the mainstay of cytotoxicity assays. Drugs need to induce cell killing if they are going to shrink the tumor. Drug concentrations and exposure times in culture should reflect and guide future administration schedules. A simple change in expression of data to “surviving cells” may go a long way to emphasizing how much better our preclinical drug development needs to be.
